# Effect of Heat Treatment on the Physical Properties of Provisional Crowns during Polymerization: An *in Vitro* Study

**DOI:** 10.3390/ma8041766

**Published:** 2015-04-15

**Authors:** May L. Mei, Sam Y. C. So, Hao Li, Chun-Hung Chu

**Affiliations:** 1 Faculty of Dentistry, the University of Hong Kong, Hong Kong, China; E-Mails: mei1123@hku.hk (M.L.M.); soyatcheong2@gmail.com (S.Y.C.S.); 2 Schools of Civil and Hydraulic Engineering, Hefei University of Technology, Hefei 230009, China; E-Mail: li.hao@hfut.edu.cn

**Keywords:** methacrylate resin, composite, provisional crown, heat treatment, flexural strength, marginal discrepancy, color stability, surface topography

## Abstract

This study concerned the effect of heat treatment during setting on the physical properties of four resin-based provisional restorative materials: Duralay (polymethyl methacrylate), Trim II (polyethyl methacrylate), Luxatemp (bis-acrylic composite), and Protemp 4 (bis-acrylic composite). Specimens were prepared at 23, 37, or 60 °C for evaluation of flexural strength, surface roughness, color change and marginal discrepancy. Flexural strength was determined by a three-point bending test. Surface profile was studied using atomic force microscopy. Color change was evaluated by comparing the color of the materials before and after placement in coffee. A travelling microscope helped prepare standardized crowns for assessment of marginal discrepancy. Flexural strength of all tested materials cured at 23 °C or 37 °C did not significantly change. The surface roughness and marginal discrepancy of the materials increased at 60 °C curing temperature. Marginal discrepancies, color stability, and other physical properties of materials cured at 23 °C or 37 °C did not significantly change. Flexural strength of certain provisional materials cured at 60 °C increased, but there was also an increase in surface roughness and marginal discrepancy.

## 1. Introduction

Fabrication of a provisional prosthesis or restoration is an essential procedure for all indirect restoration and an important stage in prosthodontics [1]. Provisional restorations protect prepared teeth, stabilize the maxillary and mandibular teeth’s relationship, address the patient’s aesthetic concerns and keep the patient comfortable from the initial tooth preparation appointment to the cementation of the permanent restoration [2]. In addition, they allow evaluation of the tooth preparation, maintain gingival health, and serve as an adjunct to periodontal therapy and as an assessment of the patient’s oral hygiene. Commonly used materials for provisional restoration are methacrylate resins and bis-acryl resin [3].

Polymethyl methacrylate (PMMA) and polyethyl methacrylate (PEMA) are methacrylate resins. They were the first available provisional methacrylate restorative material in dentistry. They stain easily due to their porosity and are color unstable. PMMA first appeared around 1940 and remains the most common material for fabrication of provisional restorations and dentures [3]. It is strong and lightweight with a high coefficient of thermal expansion. It is relatively inexpensive and capable of high polish. However, it has a strong odor, poor durability and high polymerization shrinkage.

**Figure 1:**
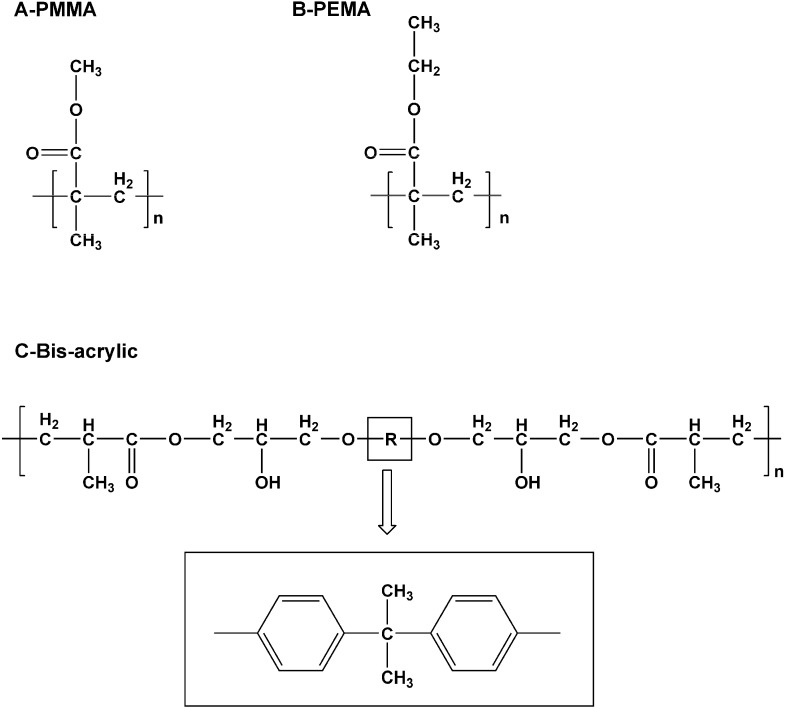
Repeating units of Polymethyl methacrylate (PMMA) (**A**); polyethyl methacrylate (PEMA) (**B**); and Bis-acrylic (**C**).

PEMA was introduced in the 1960s. An ethyl-group instead of a methyl-group was presented as the repeating unit in its molecular formula (Figure 1A,B). Although not as strong, durable, and abrasion-resistant as PMMA, it is a better selection for direct interim prosthesis fabrication [3]. It has a less pungent odor, less heat generation and less shrinkage on setting than PMMA. It is also more biocompatible and has low polymerization shrinkage [4]. Some dentists prefer it when fabricating direct provisional restoration chair side.

Bis-acrylic or bis-acrylate composite is different from methacrylate resins. It is similar to composite restorative materials because it is made of bis-acryl resin and inorganic fillers. The latter reduce polymerization shrinkage [5]. Bis-acrylic has a high strength because its monomers have a high molecular weight (Figure 1C). Compared with methacrylate resins, bis-acrylic composite has superior flexural strength and surface hardness, higher wear resistance, better marginal adaptation and lower shrinkage. However, the rigid core of the aromatic group (R in Figure 1C) makes the backbone stiff. It also prevents rotation, hindering complete polymerization [3]. Moreover, provisional bis-acrylic resin restorations for long span bridges and teeth with minimal preparation are too susceptible to fracture [6,7].

An ideal provisional restoration should be strong, durable, and adapt accurately to the margin. Therefore, the material used should resist fracture, offer a smooth, good-looking surface profile, be color-stable to resist staining from food and beverages and have an accurate marginal adaptation to protect the tooth. These properties are influenced by the curing temperature of the materials [8]. In addition, while the properties of provisional restoration depend on the type of material used, they appear to be not material specific but product specific [9]. The addition of fine particle sizes can also enhance polish ability and smoothness of the cured provisional restorative material. In clinical situations, some dentists prefer soaking the restoration into warm water to hasten curing.

This study aimed to evaluate the effect of heat treatment on the flexural strength, surface profile, color stability and marginal discrepancy of four commonly used provisional restorative materials. This study was designed to evaluate physical properties of four common commercially available provisional materials cured at 23 °C, 37 °C and 60 °C, temperatures found in the clinic, the mouth or a warm water bath. The results might help clinicians choose appropriate materials for fabricating provisional restorations for their patients.

## 2. Results and Discussion

Table 1 shows the *p*-values of two-way ANOVA main effect comparison results. Material products and curing temperature both contribute to an interaction effect on flexural strength, surface roughness, color difference, and marginal discrepancy of all the materials (*p* < 0.01, interaction *p* < 0.001). However, while color difference depended partly on product (*p* < 0.01), curing temperature did not have a main effect on color changes (*p* = 0.918).

**Table 1:** The *p*-values of main effect comparison between-subjects test of two-way ANOVA.

Factors	Flexural Strength	Surface Roughness	Color Differences	Marginal Discrepancy
Brand	<0.001	<0.001	<0.001	<0.001
Temperature	0.006	<0.001	0.918	<0.001
Brand*Temperature	<0.001	<0.001	<0.001	<0.001

### 2.1. Flexural Strength and Fracture Surface Morphology

This study used a transverse bending test to study flexural strength, also known as fracture strength or bending strength. Flexural strength is a material’s ability to resist deformation under load or, more precisely, the highest stress experienced within the material at its moment of rupture. Flexural strength is measured in terms of stress and a three-point flexural test bends a rectangular cross-section specimen until fracture or yielding [10,11]. The flexural strength of the four experimental groups is illustrated in Table 2. No significant difference of flexural strength was found in Duralay (PMMA), Trim II (PEMA) and Luxatemp (bis-acrylic composite) specimens at the three curing temperatures. The flexural strength of Protemp 4 (bis-acrylic composite) increased when cured at 60 °C. Luxatemp and Protemp 4 showed significantly higher values than Duralay and Trim II at the three curing temperatures.

**Table 2:** Flexural strength (MPa) (±SD) of the materials and curing temperatures.

Product	Materials *	A. 23 °C	B. 37 °C	C. 60 °C	Bonferroni
1. Duralay	PMMA	57.94 ± 5.37	55.82 ± 7.21	51.87 ± 9.34	NS
2. Trim II	PEMA	41.79 ± 5.37	43.61 ± 6.21	51.52 ± 5.59	NS
3. Luxatemp	BAC	106.20 ± 27.16	103.94 ± 14.15	106.91 ± 19.12	NS
4. Protemp 4	BAC	87.50 ± 10.29	89.38 ± 8.59	115.41 ± 12.76	A, B < C
Bonferroni		2 < 1 < 4 < 3	1, 2 < 3, 4	1, 2 < 3, 4	

Notes: * PMMA—Polymethyl methacrylate; PEMA—Polyethyl methacrylate; BAC—Bis-acrylic composite; NS: Not significant (*p* > 0.05).

**Figure 2:**
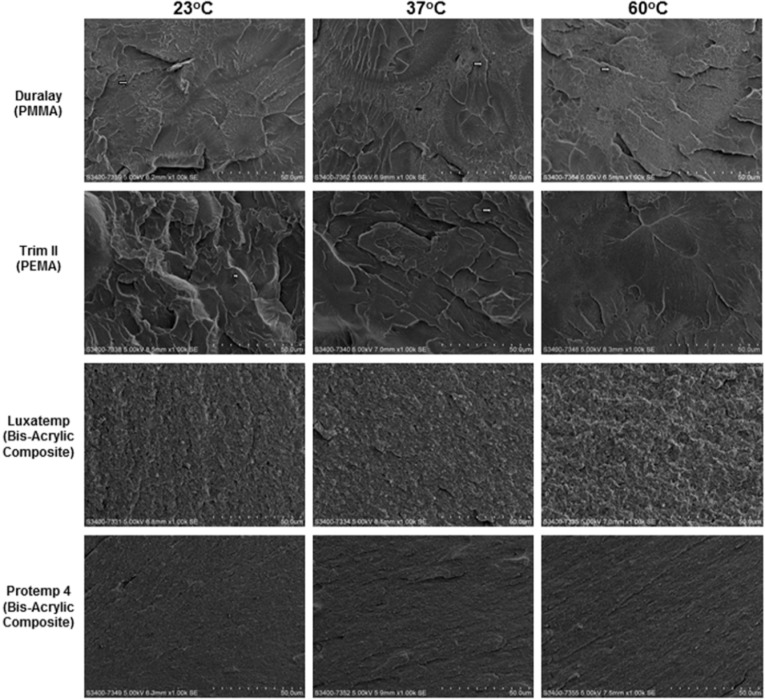
SEM images of fractured surfaces of the materials (×1000). Scale bar: 50 µm.

Figure 2 shows the morphology of the fractured surface. In Duralay and Trim II, the fractured surfaces showed step-form morphology (arrow), these steps connected at ending regions and formed a river-like structure (microcracks). These are typical morphologies of fracture through cleavage, a type of brittle fracture [12]. In Duralay, porous structures could be found at all three curing temperatures; the number of microcracks rises with increased curing temperature. The number of microcracks in Trim II reduced when temperature increased. Trim II also had a porous structure. There was less porosity in the bis-acrylic composites, Luxatemp and Protemp 4. Their fractured surfaces were flat and even, with a slight difference between different curing temperatures.

### 2.2. Surface Profile and Roughness

**Figure 3:**
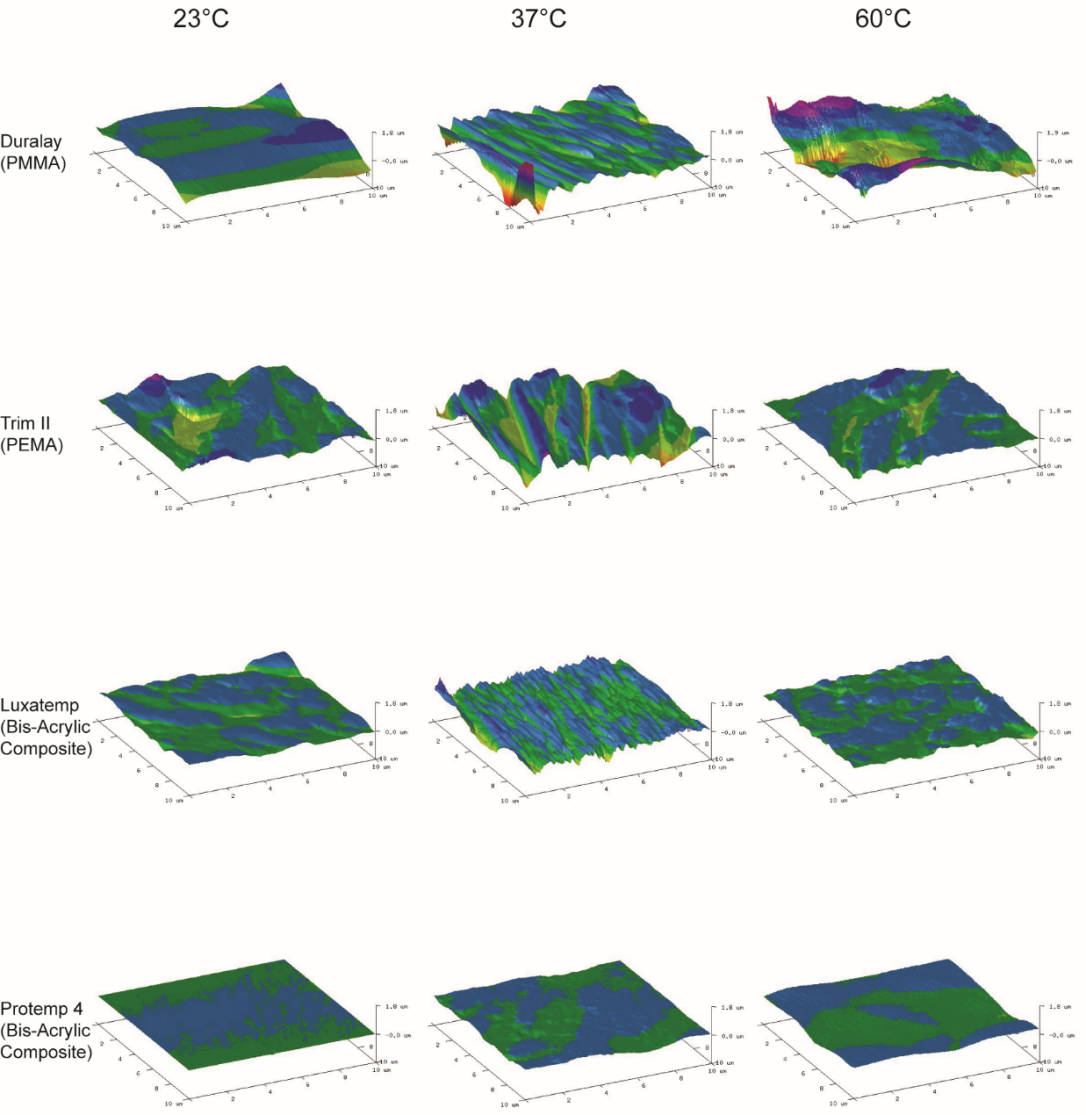
Three-dimensional Atomic Force Microscopy (AFM) tapping-mode images of the material surface.

Atomic Force Microscopy (AFM) is essential for studying surface roughness (Ra) at the nano-scale, having resolution far exceeding that of other methods, stylus-based and optical. The measured roughness of any given surface depends on the spatial and vertical resolution of the instrument. This is because real surfaces exhibit roughness on many length scales and can be thought of as a superposition of these profiles [13,14]. Table 3 shows that surfaces became rougher with curing temperature increases in all groups except for Protemp 4. Duralay curing at 60 °C has the highest Ra (180 ± 60 nm). Prompt 4 curing at 23 °C has the lowest (3 ± 0.2 nm). AFM surface profile via AFM (Figure 3) also showed a consistent result. Groups with higher Ra values showed relatively rougher surface than groups with lower Ra values. Increase in curing temperature seems to affect the Ra adversely. The studied materials cured at 60 °C had a higher Ra than those cured at 23 °C or 37 °C, except Protemp 4. Curing the composite in a warm water bath may hasten the polymerization process. However, water could also be a plasticizer, making the resin surface softer, therefore increasing the deformation during such episodes of stress [5]. Exposure to water cannot be avoided if restoration was fabricated and cured intra-orally or in a warm water bath.

**Table 3:** Surface roughness (Ra) (nm) (±SD) of the materials and curing temperatures.

Product	Materials *	A. 23 °C	B. 37 °C	C. 60 °C	Bonferroni
1. Duralay	PMMA	30 ± 14	70 ± 16	180 ± 60	A < B < C
2. Trim II	PEMA	26 ± 10	90 ± 18	100 ± 40	A, B < C
3. Luxatemp	BAC	53 ± 30	59 ± 120	110 ± 20	A, B < C
4. Protemp 4	BAC	3 ± 0.2	16 ± 2	18 ± 3	NS
Bonferroni		4 < 3	4 < 1, 2, 3	4 < 2, 3 < 1	

Notes: * PMMA—Polymethyl methacrylate; PEMA—Polyethyl methacrylat; BAC—Bis-acrylic composit; NS: Not significant (*p* > 0.05).

### 2.3. Color Stability

In esthetically critical areas, provisional restorations should provide an initially accurate color shade match and remain color-stable throughout provisional treatment [3]. The value of color change (ΔE) represents relative color change over time [15,16]. The results of ΔE are shown in Table 4. For the four provisional restorative materials tested, the highest ΔE was observed in Trim II and the lowest in Protemp 4. This matches the results of surface profile. A smoother surface results in less discoloration. A ΔE value of 3.7 or less is considered visually imperceptible and clinically acceptable [15]. In this study, ΔE was generally higher than 3.7, which could possibly be due to the long duration of the coffee soaking (28 days), with no regular tooth cleaning. This mimics an extreme situation rather than daily life. Studies indicated that PMMA resins tend to discolor less than other resins, including bis-acrylics [15,17]. However, there are also studies demonstrating that resin composite materials have better color stability than PMMA resins [18]. In this study, Protemp 4 has significantly less color change than Duralay and Trim II, and least of all at 23 °C. This finding might be related to the smoothness in Protemp 4 and low water absorption. A smooth surface can increase color stability, and absorbing much water can reduce it. Furthermore, materials with high filler content have less color change [19]. In addition to its high filler content, Protemp 4’s lower Ra and new, sophisticated fillers could cause its color stability. Curing temperature did not affect ΔE significantly in Duralay and Protemp 4.

**Table 4:** Mean color differences (ΔE*) (±SD) of the materials and curing temperatures.

Product	Materials *	A. 23 °C	B. 37 °C	C. 60 °C	Bonferroni
1. Duralay	PMMA	10.03 ± 1.76	9.86 ± 2.40	7.42 ± 3.42	NS
2. Trim II	PEMA	14.20 ± 3.06	13.77 ± 4.68	9.00 ± 4.40	A, B > C
3. Luxatemp	BAC	3.84 ± 3.17	6.33 ± 1.72	9.52 ± 1.88	A < C
4. Protemp 4	BAC	3.17 ± 0.60	2.16 ± 0.95	5.77 ± 2.10	NS
Bonferroni		4 < 3 < 1 < 2	3, 4 < 2, 4 < 1	4 < 3	

Notes: * PMMA—Polymethyl methacrylate, PEMA—Polyethyl methacrylate, BAC—Bis-acrylic composite. NS: Not significant (*p* > 0.05).

### 2.4. Marginal Discrepancy

Accurate marginal adaptation of provisional restorations is also important because it determines whether the restoration can protect the tooth from thermal, bacterial, and chemical assaults [3]. A direct technique fabricated the provisional crowns, making improper seating of the crown after polymerization inevitable, as there is no supporting abutment against the internal surface of the crown during the setting of provisional restorative material. Shrinkage toward the center of the crown will decrease the diameter of the provisional crown [20]. Table 5 shows the marginal discrepancy of experimental groups. Curing at 60 °C always has more marginal discrepancy than curing at 23 °C or 37 °C. The latter two curing temperatures show no significant difference of marginal discrepancy. However, Trim II has the most marginal discrepancy when cured at 60 °C.

**Table 5:** Marginal discrepancy (mm) (±SD) of materials and curing temperatures.

Product	Materials *	A. 23 °C	B. 37 °C	C. 60 °C	Bonferroni
1. Duralay	PMMA	0.32 ± 0.10	0.31 ± 0.06	0.42 ± 0.13	NS
2. Trim II	PEMA	0.33 ± 0.14	0.35 ± 0.09	0.77 ± 0.17	A, B < C
3. Luxatemp	BAC	0.25 ± 0.10	0.27 ± 0.04	0.58 ± 0.13	A, B < C
4. Protemp 4	BAC	0.31 ± 0.10	0.34 ± 0.08	0.49 ± 0.08	A, B < C
Bonferroni		N/A	N/A	1 < 3 < 2 4 < 2	

Notes: * PMMA—Polymethyl methacrylate; PEMA—Polyethyl methacrylate; BAC—Bis-acrylic composite; NS: Not significant (*p* > 0.05).

Ogawa *et al.* [20] studied the curing environment on mechanical properties and polymerizing behavior of methyl-methacrylate autopolymerizing resin and reported that temperature during polymerization was significant to margin fit of provisional crowns. The results of this study also showed all materials cured at 60 °C would increase marginal discrepancy between provisional restoration and prepared die (tooth). Heat activates the chemical reaction between monomer and polymer, producing a more complete polymerization and hence a greater dimensional contraction [20]. In the clinical situation, some clinicians may immerse the setting of provisional restorations in a warm water bath to shorten curing time and make restoration stronger. However, this may lead to a rougher surface, larger marginal discrepancy of the material, and obvious long-term color changes. Even though prosthesis is an accurate fit, an incorrect color leaves a patient unsatisfied [21]. The results of this study, however, did not find any significant difference in flexural strength, surface roughness, color stability, or marginal discrepancy when materials were cured at 23 °C or 37 °C. Thus, fabricating the provisional restoration on the prepared tooth *in vivo* or *ex vivo* should have no significant effect on its physical properties. The surface roughness of Duralay differed at 23 °C or 37 °C. Temperature-dependent changes in strength may differ with different products of autopolymerizing resin.

Heat activates the chemical reaction between the monomer and polymer components of the resin and facilitates a more complete polymerization [22]. This study, however, cannot corroborate this supposition. Burns *et al.* [3] proposed that the degree of polymerization stems from multiple factors. Those factors’ significance varies with the materials and is not applicable to the same material with different products. In this study, the flexural strength of PMMA (Duralay) and PEMA (Trim II) did not significantly change at 23, 37, or 60 °C. The two bis-acrylic composites (Luxatemp and Protemp 4) demonstrated significantly higher fracture strength than the two methacrylate resins (PMMA and PEMA). This might due to their lesser porosity alleviating local stress concentration and the chance of microcracks, in particular at both end regions [12]. However, the two bis-acrylic composites had their own physical behavior. Curing at 60 °C increased the facture strength of Protemp 4, but not Luxatemp. Bis-acrylic composite has fillers, unlike homogenous resins like PMMA and PEMA. This compositional difference affects mechanical properties, which could be one of the major reasons for the difference in physical behavior of the two bis-acrylic composites studied [23].

## 3. Experimental Section

This study investigated two commercially available methacrylate resin (PMMA and PEMA) and two bis-acrylic composite materials (Table 6).

**Table 6:** Provisional restorative materials used in this study.

Product	Manufacturer	Ingredient	Shade
DuraLay	Reliance Dental Mfg. Co., Chicago, IL, USA	PMMA	62
Trim II	Bosworth Co., Chicago, IL, USA	PEMA	62
Luxatemp Star	DMG, Hamburg, Germany	Bis-acrylic composites	A3
Protemp 4	3M ESPE, Seefeld, Germany	Bis-acrylic composites	A3

Specimens were chemically cured in a water bath according to their manufacturers’ instructions at 23, 37, or 60 °C. The temperatures correspond to that of *ex vivo*, *in vivo*, and warm water bath in clinical scenarios. We considered a gap of 0.3 mm as the clinically significant marginal discrepancy; and estimated the mean and the standard deviation of the width of the gaps of the tested samples at 0.2 mm and 0.1 mm, respectively. The sample size required was 10 (α = 0.05, power = 0.80).

### 3.1. Flexural Strength and Fracture Surface Morphology

Ten rectangular specimen beams in each group with the dimensions of 25 × 2 × 2 mm^3^ were fabricated with a custom-made stainless steel mold. The specimens were then thermocycled in distilled water at temperatures of 5 °C and 55 °C for 3000 cycles, with a dwell time of 20 s in each water bath to simulate *in vitro* thermal changes that occur in the oral cavity [24]. After the thermocycling, a three-point bending test with a universal testing machine (ElectroPulsTM E3000, Instron, Norwood, MA, USA) determined the flexural strength of each specimen. A load weighed on the specimen surface at a crosshead speed of 1 mm/min until failure. The following equation calculated flexural strength (*F*):

*F = 3PL/2bh*^2^ (1)

*P* was the load at fracture; *L* the test span; *b* the thickness of the sample; *h* the height of the sample. Fractographic examination was performed to assess the cause of failure by studying the characteristics of the fracture surface. Scanning electron microscopy (SEM) (Hitachi S-3400 FEG Scanning Electron Microscope, Hitachi Ltd., Tokyo, Japan) at 5 kV in high-vacuum mode revealed the fracture surface morphologies of the specimens.

### 3.2. Surface Profile and Roughness

Atomic force microscopy (AFM) (Dimension Edge, Bruker, CA, USA) was used to evaluate the surface topography and surface roughness of experimental materials. Three rectangular specimens were from each group were prepared to 5 × 5 × 2 mm^3^ using a custom-made Teflon mould followed by polishing to 4000-grit using a polisher (Ecomet 6 variable speed polisher, Buehler, IL, USA) before testing. AFM analysis was conducted using a tapping model etched silicon probe. Images were analyzed using NanoScope Analysis 1.40 (Bruker, CA, USA) and 3D images were normalized in scale Z [25]. Surface roughness was defined as the arithmetical average of the surface height relative to the mean height (Ra). A high value of Ra represents a rough surface [26]. Ten areas of 2 × 2 µm^2^ were randomly chosen from upper, middle, lower, left and right areas in each specimen for evaluation and a mean value of Ra was calculated for each specimen.

### 3.3. Color Stability

The specimens used in the Ra test then went through a color stability test, involving coffee. The test dissolved 20 g of coffee powder (Café extra forte, Melitta, Brazil) into 1000 mL of boiled distilled water, stirring every 30 min for 10 s until it cooled to 37 °C, then filtered it through a filter paper (0.6 μm, Whatman^®^, Buckinghamshire, UK). The specimens were immersed in the coffee solution and kept at 37 °C in an incubator. The coffee solution was replaced every 24 h. Color stability was evaluated by comparing the color of the specimens before and after the incubation in the coffee solution at 37 °C for 28 days. Each specimen was fixed on a stand with standard lighting units (Kaiser RB2 Lighting Unit, Kaiser Fototechnik, London, UK) for color assessment. A fixed lens (focal length: 50 mm, F-Stop: 5.6, ISO Speed Ratings: 400) took images of the specimens mounted on top of the stand. The images were imported into Photoshop CS (Adobe Systems, San Jose, CA, USA). Measurements were taken at the top, middle and bottom area of each. White balance was calibrated according to a white calibration standard. Color changes were characterized using the Commission Internationale d’Eclairge L*a*b* color space (CIELAB) [15]. Parameter L* represents the degree of grey and corresponds to lightness. Parameter a* represents the red-green axis, whereas b* is a parameter for the blue-yellow axis. Total color differences are expressed by the formula

(2) $$∆E=\sqrt{{\left({L^{*}}_{f}-{L^{*}}_{i}\;\right)}^{2}+{\left({a^{*}}_{f}-{a^{*}}_{i}\;\right)}^{2}+{\left({b^{*}}_{f}-{b^{*}}_{i}\;\right)}^{2}}$$

where the initial (*i*) and final (*f*) are color descriptors for before and after coffee immersion.

### 3.4. Evaluation of Marginal Discrepancy

The method for evaluation of marginal discrepancy was adopted from a previous study by Balkenhol *et al.* [14,25]. The laboratory procedures of fabricating the specimen crown for evaluation simulated chair side fabrication of provisional restoration. The first stage in fabricating the provisional crowns involved taking a vinyl polysiloxane impression (Imprint 3, 3M ESPE, St Paul, MN, USA) of the “normal teeth” dies, which were fabricated as premolar size with a diameter of 8 mm and a 3° taper to the vertical axis. Then they were replaced with the “prepared teeth” dies of 5 mm in diameter. There was 1.5 mm width shoulder representing shoulder preparation. The height was 8.5 mm, equivalent to uniform reduction of tooth structure circumferentially and occlusally by 1.5 mm [27]. The “prepared teeth” dental dies also tapered at an angle of 3°. The provisional restoration material was dispensed into the impression, which was then placed back to the test model with “prepared teeth” dies, under a load of 500 g. The impression was removed from the model after initial setting. The crown was soaked into a water bath for 1 min at 23, 37, or 60 °C. Ten crowns were prepared for each provisional restoration material and temperature. The polymerized crowns were replaced on “prepared teeth” dies by exerting a force of about 100 g using a dead weight of 100 g. The test model was tilted through 90° so that the margin of provisional crowns could be determined on two opposite sides of the dies. The measurement points were selected every 60° of circumference around the crown’s marginal line, using a traveling microscope (Leitz WetzLar, Wetzlar, Germany) at 30× magnification.

### 3.5. Statistical Analysis

The Shapiro-Wilk test was used to test the normality of the data. Two-way analysis of variance (ANOVA) with main effect compared the effects of different materials and curing temperatures (as two predicting variables) on flexural strength, surface roughness, color change and marginal discrepancy of the specimens. Bonferroni adjustment for multiple testing reduced the chance of type I error. The computer software SPSS Statistics—Version 20.0 (IBM Corporation, Armonk, NY, USA) performed analysis. The level of statistical significance for all tests was 0.05.

## 4. Conclusions

Flexural strength of bis-acrylic composite provisional materials cured at 60 °C increased, but there is also an increase in surface roughness and marginal discrepancy. Apart from the surface roughness of bis-acrylic composite provisional materials, curing at 23 °C or 37 °C does not significantly alter the physical properties of the four resin-based provisional restorative materials. Within the limitation of this study, it is suggested that the fabrication of provisional crown restorations *in vivo* does not have a significant effect on their properties. Immersing the provisional crown materials in warm water bath may strengthen them but it increases their marginal discrepancy, roughens their surfaces and reduces their color stability.
